# Physical Orthopædics

**Published:** 1917-09-01

**Authors:** Fred French

**Affiliations:** Northampton


### Physical Orthopædics.

*To the Editor of* The Hospital.

Sir,—I have read Dr. Fortescue Fox's article in The Hospital, which I take regularly, and am much interested in what he says therein. He is quite right when he states that the lack of natural methods of treatment, such as Baths, Massage, Remedial Exercises, Orthopædics, etc., is, certainly a grave national deficiency. As one who has had to practice Massage and Orthopædics for some twelve years and who is practising both in Wounded and Accident Hospitals, I have long felt that all these branches should be taught in recognised institutions and thus be classed as proper methods of Curative Treatment.

We know, and have had it strikingly illustrated since the war began, that (1) Diet, (2) Physical Culture, (3) Orthopædics, (4) Hydropathy, (5) Remedial Gymnastics, (6) Massage, (7) Bone-setting, and even (8) Chiropody have all proved invaluable, both in the preparation of recruits and in the treatment of the disabled crippled and wounded. Now is it not a fact that there is not a recognised College for the scientific training of any of these branches of curative work in the country? True, there is an Institute for Masseuses, and, I believe, an Institute for Chiropody, and there are two or three Physical Culture Colleges, but they are only isolated and not national institutions, and I doubt if they are medically recognised as Preventive or Curative Treatment Institutions. Take the question of Orthopædics, i e. the art of curing deformities, and of Medical Gymnastics. Of the many hundreds of thousands (probably about three millions would be nearer the mark) of men, women, and children with physical deformities, the vast majority are curable, and yet the bare fact is that there are not as many experts in the country as can be counted on the fingers of the two hands! The same applies to Medical Gymnastics for Digestive, Nervous, Circulatory troubles, Debility, Convalescence, etc. Hundreds of thousands of these cases are advised Remedial Exercises, but how many trained experts are there to give them, and how many Colleges and Institutes are there where they may be trained. Pigeon-chest and contracted chest are deformities that literally hundreds of thousands of children suffer from in this country, and there is not a single Institution where they are treated!

I began to study these defects in the Lancashire cotton-operatives' children several years ago, and was even then amazed at the immense amount of chest and other physical defect present, and after years of investigation and practical application I elaborated a system of treatment by means of deep-breathing movements, chest-expander movements, and graduated weights to place on the sternum in the case of pigeon-chest; and of breathing and "drawing-out" exercises in the case of contracted chest; but there was no institute in England—or in any other country—where I could have learnt it. Sweden is at least fifty years ahead of us in the way of medical gymnastics, massage, and orthopædics, and so is Finland. In these countries for many years schoolchildren and adults have been treated for physical defects and ailments, including incipient phthisis, in conjunction with the education authorities in the case of the children. They are also in close touch with the hospitals for cases of after-treatment in the case of injuries, a speciality being made of fracture treatment. The gymnastic orthopædists, as they are called, not only work in close conjunction with the medical men, but in many cases they are both doctor and medical orthopædist combined, and both have an equal status there; and, further, both are State professions. The medical gymnasts, masseurs, and orthopædists here are isolated, and work more or less on their own, picking up their patients with or without medical advice. As I stated before, there is no State or medically recognised institution whereby they can become qualified; therefore, I would ask, Is it fair and right to dub any of these (many having graduated in the school of many years' actual experience and practice, which is, after all, the real thing) to be incompetent and unqualified?

Let us have as soon as possible a College of Medical Gymnastics (which will include medical exercise, massage, orthopædics, bone-setting, hydropathy, etc.), or call the College some comprehensive name. Let all these treatments be placed on a proper basis, and we shall have no lack of qualified experts because there will be then the proper places to qualify in. Not only for the present wounded soldier and half-cured discharged man, who in their tens of thousands are urgently in need of treatment, but the multitudes of civilians, adults and children, whose deformity, paralysis, crippled weakly and weedy children call aloud for, and will call louder and louder for, treatment by these methods. The medical man and the expert in physical treatment will then work hand in hand: at present all is chaos.—Yours obediently,

Northampton, August 18, 1917. Fred. French.

